# Supplementary material for: IgG and IgM cooperate in coating of intestinal bacteria in IgA deficiency
Source: Nat Commun. 2023 Dec 8;14:8124. doi: 10.1038/s41467-023-44007-2 (PMC10709418; doi:10.1038/s41467-023-44007-2)
Supplement: Supplementary file 1 — Supplementary Information [file 41467_2023_44007_MOESM1_ESM.pdf]

IgG and IgM cooperate in coating of intestinal bacteria in IgA deficiency

Carsten Eriksen[1,2], Janne Marie Moll[1], Pernille Neve Myers[1], Ana Rosa Almeida Pinto[1], Niels Banhos Danneskiold-Samsøe[3], Rasmus Ibsen Dehli[1], Lisbeth Buus Rosholm[1], Marlene Danner Dalgaard[4], John Penders[5], Daisy MAE Jonkers[6], Qiang Pan-Hammarström[7], Lennart Hammarström[8], Karsten Kristiansen[2,3,9,10], Susanne Brix[1,2*]

[1]Department of Biotechnology and Biomedicine, Technical University of Denmark, Kgs. Lyngby, Denmark; [2]Center for Molecular Prediction of Inflammatory Bowel Disease, Department of Clinical Medicine, Aalborg University, Copenhagen, Denmark; [3]Laboratory of Genomics and Molecular Biomedicine, Department of Biology, University of Copenhagen, Copenhagen, Denmark; [4]Department of Health Technologies, Technical University of Denmark, Kgs. Lyngby, Denmark; [5]Department of Medical Microbiology, Infectious Diseases and Infection Prevention, NUTRIM School for Nutrition and Translational Research in Metabolism & Care and Public Health Research Institute CAPHRI, Maastricht University Medical Centre, Maastricht, The Netherlands; [6]Division Gastroenterology-Hepatology, Department of Internal Medicine, NUTRIM School for Nutrition and Translation Research in Metabolism, Maastricht University Medical Centre+, Maastricht, The Netherlands;[7]Department of Biosciences and Nutrition, Karolinska Institute, Stockholm, Sweden; [8]Department of Laboratory Medicine, Karolinska Institute, Stockholm, Sweden; [9]BGI-Shenzhen, Shenzhen 518083, China; [10]Qingdao-Europe Advanced Institute for Life Sciences, Qingdao, Shandong 266555, China.

*Corresponding author: Susanne Brix, Department of Biotechnology and Biomedicine, Soltofts Plads, Building 224, Technical University of Denmark, DK-2800 Kgs. Lyngby, Denmark, sbrix@dtu.dk, (+45) 21604264

Supplementary Figures

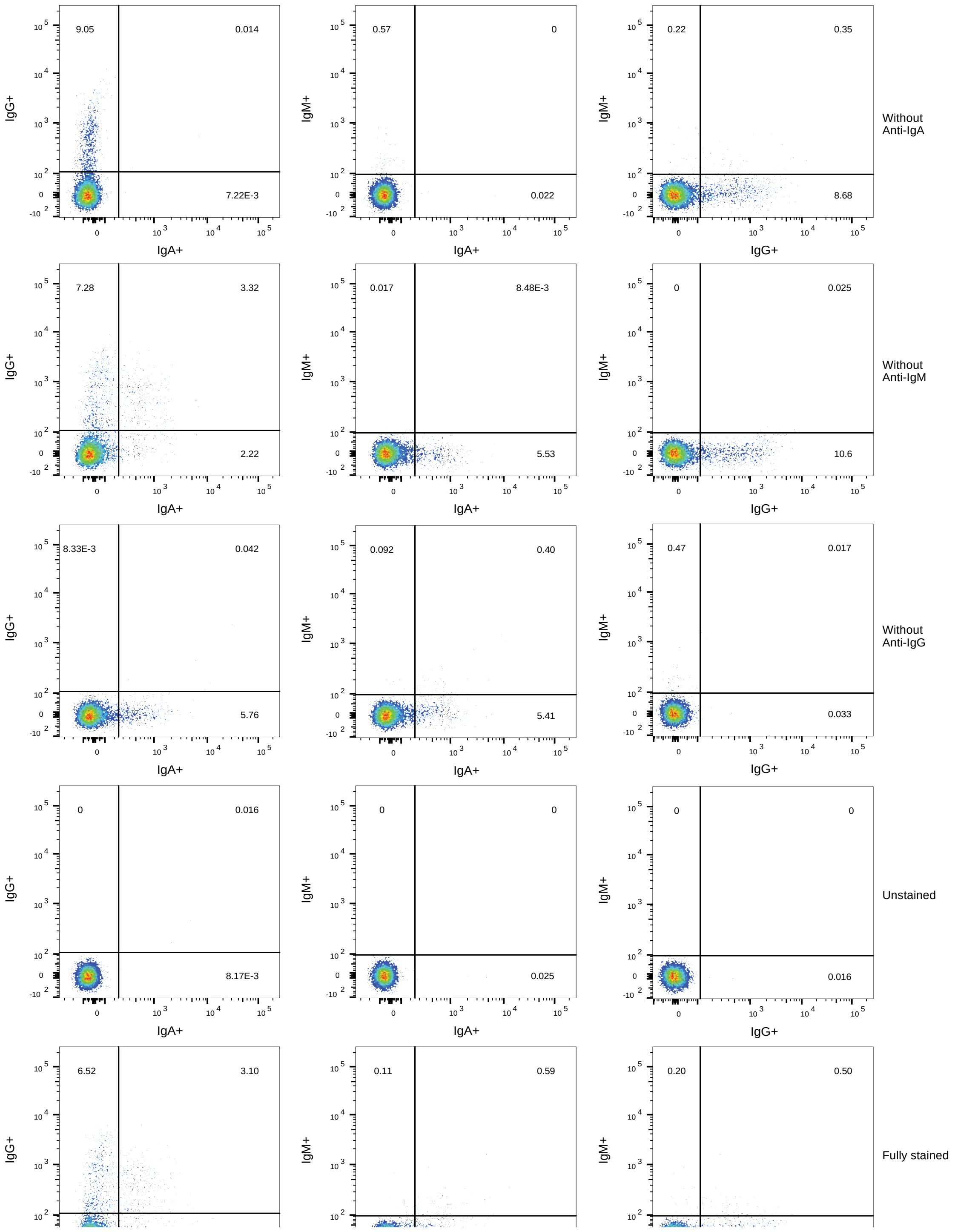

Supplementary Fig. 1. Flow cytometry-based gating of IgA, IgG and IgM-coated bacteria including fluorescence-minus-one controls. Fluorescence-minus-one (FMO) controls for the IgA, IgM and IgG stainings. Performed on fecal samples from IgA+ subjects.

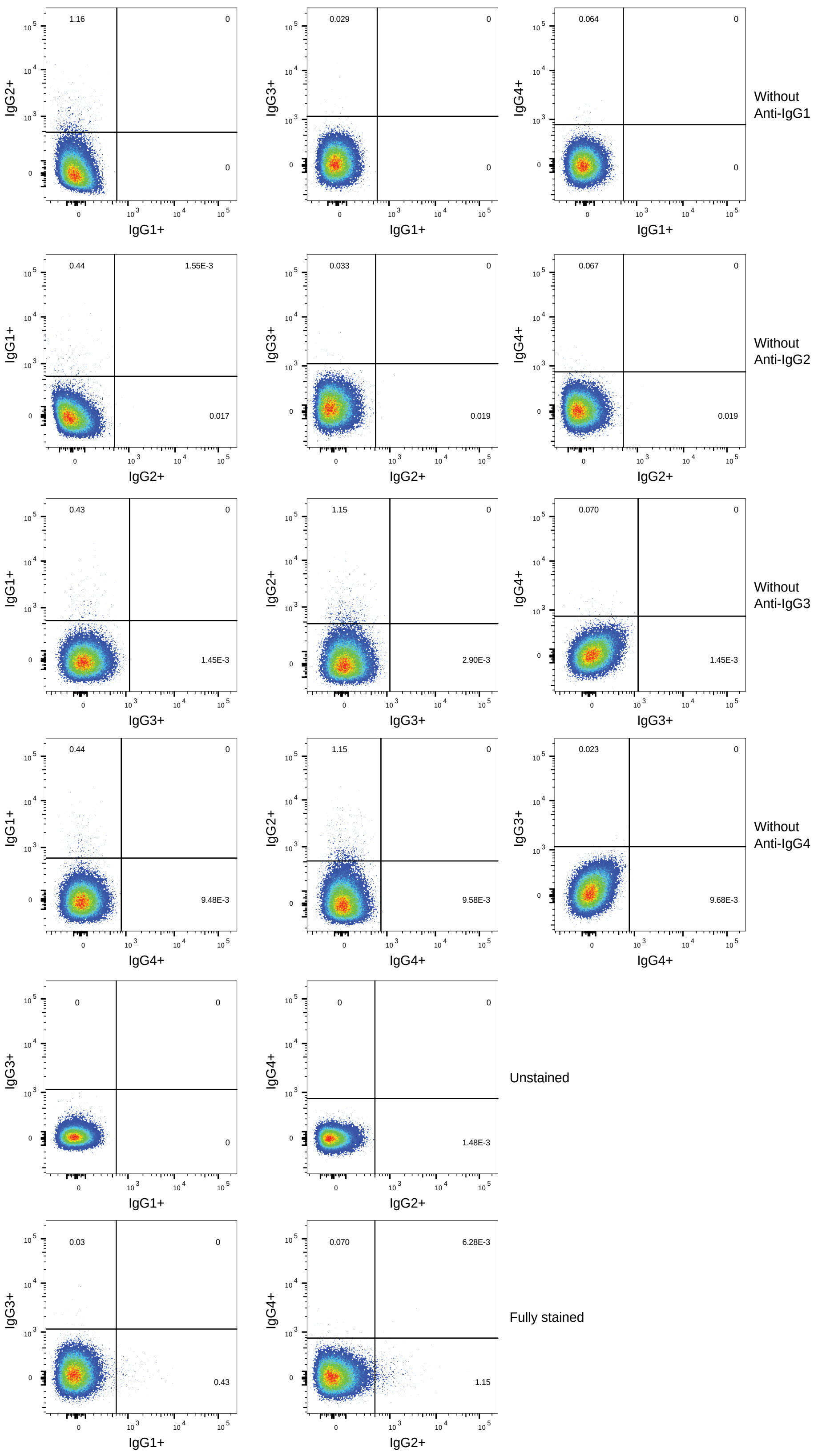

Supplementary Fig. 2. Flow cytometry-based gating of IgG1-4 subtype coated bacteria including FMO controls. FMO controls for the four IgG subtype stainings. Performed on a mixture of five fecal samples.

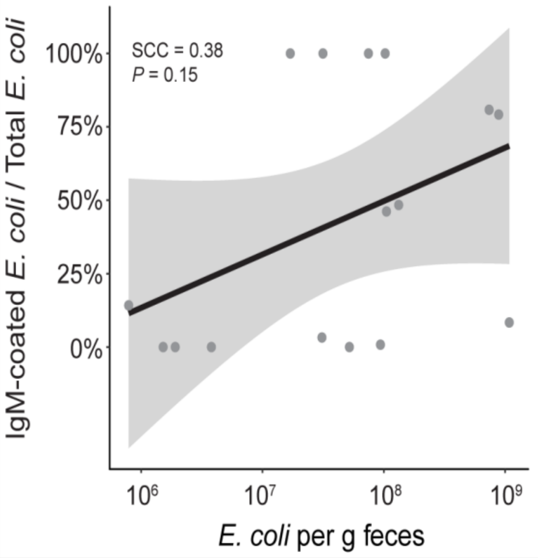

Supplementary Fig. 3. Association of fecal load of *E. coli* to the fraction of IgM-coated *E. coli*. Spearman rank correlation between the fecal load of *E. coli* and (a) the fraction of IgM-coated *E. coli* (n=16). Rho and *P* values are provided in the plot. The straight line and the shaded area indicate the parametric correlation coefficient and the 95% confidence interval, respectively.

Supplementary Tables

Table S1 - Median abundance of serum immunoglobulins and Ig-coated in % or as bacteria/g feces in IgA- (n = 31) and IgA+ (n = 31) subjects. Differences are tested by two-sided Wilcoxon.

	**IgA- subjects**		**IgA+ subjects**					
	Mean [SD]	Median [25th; 75th quantile]	Mean [SD]	Median [25th; 75th quantile]	***P*-value**	**FDR adjusted**	**Significance**	**Method**
Bacterial load (bacteria/g feces)	2.93E+10 [1.25E+10]	2.69E+10 [2.14E+10; 3.71E+10]	3.31E+10 [2.08E+10]	2.63E+10 [2.05E+10; 4.06E+10]	8.00E-01	8.00E-01	no	Wilcoxon (two-sided)
Serum IgM (g/L)	1.03 [0.72]	0.78 [0.61; 1.16]	0.90 [0.48]	0.86 [0.59; 1.06]	7.85E-01	7.80E-01	no	Wilcoxon (two-sided)
Serum IgG (g/L)	15.77 [3.57]	15.7 [13.75; 17.70]	10.19 [2.04]	9.41 [9.07; 10.85]	1.47E-04	**2.90E-04**	**yes**	Wilcoxon (two-sided)
IgA (% coated bacteria)	0 [0]	0 [0; 0]	9.28 [4.59]	8.93 [6.40; 11.29]	5.04E-13	**1.50E-12**	**yes**	Wilcoxon (two-sided)
IgM (% coated bacteria)	3.68 [4.97]	2.18 [0.71; 4.19]	0.92 [1.48]	0.17 [0.05; 1.10]	1.03E-04	**1.50E-04**	**yes**	Wilcoxon (two-sided)
IgG (% coated bacteria)	1.74 [1.51]	1.17 [0.73; 2.35]	2.50 [2.03]	2.21 [1.00; 3.42]	1.09E-01	1.09E-01	no	Wilcoxon (two-sided)
IgA (bacteria/g feces)	0 [0]	0 [0; 0]	2.77E+09 [1.63E+09]	2.38E+09 [1.84E+09; 3.71E+09]	5.05E-13	**5.10E-13**	**yes**	Wilcoxon (two-sided)
IgM (bacteria/g feces)	1.14E+09 [1.81E+09]	5.12E+08 [1.89E+08; 1.11E+09]	2.31E+08 [3.67E+08]	6.76E+07 [1.45E+07; 3.00E+08]	1.80E-04	**1.20E-04**	**yes**	Wilcoxon (two-sided)
IgG (bacteria/g feces)	5.41E+08 [5.32E+8]	3.10E+08 [1.86E+08; 7.83E+08]	7.24E+08 [6.14E+8]	6.44E+08 [2.07E+08; 9.95E+08]	1.90E-01	1.90E-01	no	Wilcoxon (two-sided)

Table S2 - Median abundance of single and multi IgA, IgG and/or IgM coated bacteria/g feces in IgA- (n = 31) and IgA+ (n = 31) subjects. Differences are tested by two-sided Wilcoxon.

	**IgA- subjects**		**IgA+ subjects**					
	Mean [SD]	Median [25th; 75th quantile]	Mean [SD]	Median [25th; 75th quantile]	***P*-value**	**FDR adjusted**	**Significance**	**Method**
Single-IgA (bacteria/g feces)	0 [0]	0 [0; 0]	2.12E+09 [1.41E+09]	1.47E+09 [1.06E+09; 3.23E+09]	5.05E-13	**3.50E-12**	**yes**	Wilcoxon (two-sided)
Single-IgM (bacteria/g feces)	8.18E+08 [1.66E+09]	2.18E+08 [5.86E+07; 5.89E+08]	1.27E+07 [1.81E+07]	0 [0; 2.27E+07]	4.79E-08	**1.10E-07**	**yes**	Wilcoxon (two-sided)
Single-IgG (bacteria/g feces)	2.18E+08 [2.36E+08]	1.56E+08 [3.19E+07; 3.09E+08]	8.67E+07 [1.28E+08]	5.05E+07 [2.76E+07; 9.45E+07]	4.55E-02	**4.50E-02**	**yes**	Wilcoxon (two-sided)
IgAIgG (bacteria/g feces)	0 [0]	0 [0; 0]	4.51E+08 [3.82E+08]	3.17E+08 [1.53E+08; 7.83E+08]	1.91E-12	**6.70E-12**	**yes**	Wilcoxon (two-sided)
IgAIgM (bacteria/g feces)	0 [0]	0 [0; 0]	3.24E+07 [7.12E+07]	0 [0; 2.95E+07]	6.82E-04	**8.00E-04**	**yes**	Wilcoxon (two-sided)
IgGIgM (bacteria/g feces)	3.22E+08 [3.71E+08]	1.76E+08 [3.01E+07; 5.26E+08]	3.01E+07 [1.04E+08]	0 [0; 0]	1.23E-07	**2.10E-07**	**yes**	Wilcoxon (two-sided)
IgAIgGIgM (bacteria/g feces)	0 [0]	0 [0; 0]	1.55E+08 [2.67E+08]	5.34E+07 [0; 1.78E+08]	3.98E-07	**5.60E-07**	**yes**	Wilcoxon (two-sided)

Table S3: Mean IgA and IgM sorted bacteria/g feces at family level (n = 16 of each). Differential abundance calculated by two-sided Wilcoxon.

Phylum	Family	Mean (IgA+ Sorted)	SD (IgA+ Sorted)	Mean (IgM+ Sorted)	SD (IgM+ Sorted)	*P* -value	FDR adjusted	Significance	Method
Actinobacteria	Actinomycetaceae	0.00E+00	0.00E+00	1.16E+05	4.62E+05	1.00E+00	1.00E+00	no	Wilcoxon (two-sided)
Actinobacteria	Bifidobacteriaceae	1.10E+07	2.34E+07	2.25E+07	6.95E+07	7.82E-01	9.30E-01	no	Wilcoxon (two-sided)
Actinobacteria	Eggerthellaceae	5.25E+03	2.10E+04	1.35E+06	4.16E+06	5.92E-02	4.60E-01	no	Wilcoxon (two-sided)
Bacteroidetes	Bacteroidaceae	1.23E+06	3.71E+06	1.06E+08	4.19E+08	1.44E-01	4.60E-01	no	Wilcoxon (two-sided)
Bacteroidetes	Barnesiellaceae	2.70E+05	1.08E+06	0.00E+00	0.00E+00	1.00E+00	1.00E+00	no	Wilcoxon (two-sided)
Bacteroidetes	Muribaculaceae	6.13E+05	2.45E+06	3.14E+05	1.25E+06	8.55E-01	9.60E-01	no	Wilcoxon (two-sided)
Bacteroidetes	Prevotellaceae	1.40E+07	4.85E+07	8.74E+06	3.50E+07	9.33E-01	9.70E-01	no	Wilcoxon (two-sided)
Bacteroidetes	Rikenellaceae	1.04E+05	3.41E+05	1.79E+05	4.16E+05	1.06E-01	4.60E-01	no	Wilcoxon (two-sided)
Firmicutes	Acidaminococcaceae	9.55E+06	3.56E+07	5.40E+06	1.66E+07	9.69E-01	9.70E-01	no	Wilcoxon (two-sided)
Firmicutes	Christensenellaceae	1.31E+08	3.97E+08	2.08E+07	6.89E+07	3.48E-01	6.40E-01	no	Wilcoxon (two-sided)
Firmicutes	Clostridiaceae	1.99E+07	4.83E+07	6.09E+06	1.70E+07	3.75E-01	6.40E-01	no	Wilcoxon (two-sided)
Firmicutes	Clostridiales Family XIII	2.78E+06	9.14E+06	3.96E+06	1.52E+07	6.25E-01	9.10E-01	no	Wilcoxon (two-sided)
Firmicutes	Enterococcaceae	0.00E+00	0.00E+00	5.19E+06	2.08E+07	1.00E+00	1.00E+00	no	Wilcoxon (two-sided)
Firmicutes	Erysipelotrichaceae	2.26E+07	7.89E+07	5.38E+06	1.77E+07	1.44E-01	4.60E-01	no	Wilcoxon (two-sided)
Firmicutes	Lachnospiraceae	6.55E+08	1.68E+09	6.85E+08	1.99E+09	7.82E-01	9.30E-01	no	Wilcoxon (two-sided)
Firmicutes	Peptococcaceae	0.00E+00	0.00E+00	7.49E+04	3.00E+05	1.00E+00	1.00E+00	no	Wilcoxon (two-sided)
Firmicutes	Peptostreptococcaceae	4.92E+08	1.20E+09	9.91E+07	3.05E+08	1.75E-01	4.80E-01	no	Wilcoxon (two-sided)
Firmicutes	Ruminococcaceae	8.26E+08	1.95E+09	6.37E+08	1.86E+09	1.05E-01	4.60E-01	no	Wilcoxon (two-sided)
Firmicutes	Streptococcaceae	6.19E+06	1.54E+07	1.54E+07	4.50E+07	7.54E-01	9.30E-01	no	Wilcoxon (two-sided)
Firmicutes	Veillonellaceae	1.96E+07	6.63E+07	7.79E+06	2.12E+07	3.49E-01	6.40E-01	no	Wilcoxon (two-sided)
Proteobacteria	Desulfovibrionaceae	1.04E+06	4.15E+06	0.00E+00	0.00E+00	1.00E+00	1.00E+00	no	Wilcoxon (two-sided)
Proteobacteria	Enterobacteriaceae	1.80E+06	6.01E+06	8.38E+07	2.21E+08	**9.72E-03**	1.80E-01	yes	Wilcoxon (two-sided)
Verrucomicrobia	Akkermansiaceae	3.47E+08	1.16E+09	1.75E+08	5.91E+08	2.93E-01	6.40E-01	no	Wilcoxon (two-sided)

Table S4 - Median abundance of single and multi IgG1-4 coated bacteria/g feces in IgA- (n = 31) and IgA+ (n = 31) subjects. Differences are tested by two-sided Wilcoxon.

	IgA- subjects	IgA+ subjects				
	**Median [25th; 75th quantile]**	**Median [25th; 75th quantile]**	***P*-value**	**FDR adjusted**	**Significance**	**Method**
IgG1 (bacteria/g feces)	1.84E+08 [9.30E+07; 2.83E+08]	1.44E+08 [8.40E+07; 2.47E+08]	5.64E-01	6.80E-01	no	Wilcoxon (two-sided)
IgG2 (bacteria/g feces)	0 [0; 4.47E+06]	0 [0; 0]	1.72E-01	6.80E-01	no	Wilcoxon (two-sided)
IgG3 (bacteria/g feces)	0 [0; 0]	0 [0; 0]	6.76E-01	6.80E-01	no	Wilcoxon (two-sided)
IgG4 (bacteria/g feces)	1.19E+08 [6.39E+07; 1.80E+08]	1.03E+08 [5.98E+07; 1.40E+08]	5.17E-01	6.80E-01	no	Wilcoxon (two-sided)
Single-IgG1 (bacteria/g feces)	1.55E+08 [8.48E+07; 2.64E+08]	1.44E+08 [8.40E+07; 2.44E+08]	8.33E-01	8.30E-01	no	Wilcoxon (two-sided)
Single-IgG2 (bacteria/g feces)	0 [0; 1.76E+06]	0 [0; 0]	3.04E-01	5.40E-01	no	Wilcoxon (two-sided)
Single-IgG3 (bacteria/g feces)	0 [0; 0]	0 [0; 0]	3.05E-01	5.40E-01	no	Wilcoxon (two-sided)
Single-IgG4 (bacteria/g feces)	1.06E+08 [6.30E+07; 1.76E+08]	9.81E+07 [5.98E+07; 1.36E+08]	5.26E-01	6.70E-01	no	Wilcoxon (two-sided)
IgG1IgG2 (bacteria/g feces)	0 [0; 0]	0 [0; 0]	3.58E-01	5.40E-01	no	Wilcoxon (two-sided)
IgG1IgG3 (bacteria/g feces)	0 [0; 0]	0 [0; 0]	5.96E-01	6.70E-01	no	Wilcoxon (two-sided)
IgG1IgG4 (bacteria/g feces)	0 [0; 9.55E+06]	0 [0; 1.52E+06]	1.55E-01	5.40E-01	no	Wilcoxon (two-sided)
IgG2IgG4 (bacteria/g feces)	0 [0; 0]	0 [0; 0]	1.61E-01	5.40E-01	no	Wilcoxon (two-sided)
IgG1IgG2IgG4 (bacteria/g feces)	0 [0; 0]	0 [0; 0]	3.33E-01	5.40E-01	no	Wilcoxon (two-sided)

Table S5: Spearman correlations (two-sided) between *E. Coli* and Ig-coating

Donor type	Species	Isotype	SCC	*P*-value	FDR adjusted	Significance	Method
IgA-	*Escherichia coli*	IgGIgM	0.69	1.59E-05	1.43E-04	**yes**	Spearman correlation
IgA-	*Escherichia coli*	Single IgG	0.25	1.72E-01	3.88E-01	no	Spearman correlation
IgA-	*Escherichia coli*	Single IgM	0.35	5.56E-02	1.67E-01	no	Spearman correlation
IgA+	*Escherichia coli*	IgAIgG	-0.03	8.57E-01	9.32E-01	no	Spearman correlation
IgA+	*Escherichia coli*	IgAIgM	-0.15	4.23E-01	5.44E-01	no	Spearman correlation
IgA+	*Escherichia coli*	IgGIgM	-0.02	9.32E-01	9.32E-01	no	Spearman correlation
IgA+	*Escherichia coli*	Single IgA	0.46	8.47E-03	3.81E-02	**yes**	Spearman correlation
IgA+	*Escherichia coli*	Single IgG	0.15	4.18E-01	5.44E-01	no	Spearman correlation
IgA+	*Escherichia coli*	Single IgM	-0.16	4.03E-01	5.44E-01	no	Spearman correlation

Donor type	Species	Isotype	SCC	*P*-value	FDR adjusted	Significance	Method
IgA-	*Escherichia coli*	Single IgG1	0.24	2.09E-01	2.35E-01	no	Spearman correlation
IgA-	*Escherichia coli*	Single IgG2	0.76	3.68E-02	6.62E-02	**yes**	Spearman correlation
IgA-	*Escherichia coli*	Single IgG4	0.33	7.85E-02	1.18E-01	no	Spearman correlation
IgA+	*Escherichia coli*	Single IgG1	0.44	2.76E-02	6.22E-02	**yes**	Spearman correlation
IgA+	*Escherichia coli*	Single IgG2	0.80	3.33E-01	3.33E-01	no	Spearman correlation
IgA+	*Escherichia coli*	Single IgG4	0.33	1.06E-01	1.36E-01	no	Spearman correlation
All donors	*Escherichia coli*	Single IgG1	0.33	1.49E-02	6.09E-02	**yes**	Spearman correlation
All donors	*Escherichia coli*	Single IgG2	0.78	4.66E-03	4.19E-02	**yes**	Spearman correlation
All donors	*Escherichia coli*	Single IgG4	0.32	2.03E-02	6.09E-02	**yes**	Spearman correlation

Supplementary Materials and Methods

DNA extraction and 16S rRNA library preparation from flow cytometry-sorted samples

Bacterial DNA was extracted using the NucleoSpin Soil kit (Macherey-Nagel, Germany) based on the manufacturer's protocol. The extracted DNA was amplified using a two-step PCR reaction with the 314F (TCGTCGGCAGCGTCAGATGTGTATAAGAGACAGCCTACGGGNGGCWGCAG) and 806R (GTCTCGTGGGCTCGGAGATGTGTATAAGAGACAGGACTACHVGGGTATCTAATCC) targeting the hypervariable regions V3+V4 of the 16S ribosomal RNA gene. The DNA was amplified using the Phusion Green High-Fidelity DNA Polymerase kit (Thermo Fisher). For PCR, master mix was added to 20 µL extracted DNA in concentrations according to manufacturer's recommendations. The PCRs were performed using the following conditions: initial denaturation for 30s at 98°C followed by 28 cycles of 10s 98°C, 15s 56°C and 30s 72°C with a final elongation at 72°C for 5 min. The amplicons were tagged with Illumina adapters (Forward: AATGATACGGCGACCACCGAGATCTACAC, Reverse: CAAGCAGAAGACGGCATACGAGAT) using 10 cycles of the same PCR conditions. Both amplicons were purified after each PCR using Agencourt AMPure XP beads (Beckman Coulter). In preparation for sequencing, similar volumes of all amplicons were pooled and the library was diluted to a total concentration of 4 nM before sequencing on the MiSeq platform (Illumina, USA) using the v3 kit (paired-end).

16S rRNA Gene Data Processing

Sequencing adapters were removed using the BBDuk tool in the BBTools package v38.37 (BBDuk - Bushnell B. - sourceforge.net/projects/bbmap/). Reads were analyzed and denoised using DADA2 v. 1.10[1]. Resulting amplicon sequence variants (ASVs), representing uniquely identified bacteria, were mapped to the 99% identity clustered SILVA database v132[2] using a naive Bayes classifier[3] trained on the amplified region, as implemented in DADA2.

Several control samples were collected during the workflow: pre-sorting sheath fluid collected at the outlet from the FACS sorter (one per sorting day), blank DNA extraction controls (one per extraction day), and buffer controls for each PCR reaction. We corrected for reagent and pre-sorting fluid contamination by subtracting the bacterial read counts found in the above controls that corresponded to the respective sorting day, DNA extraction day and library preparation. Please consult **Table S3** for all read data.

After correction for contaminating reads and filtering out very low-abundant ASVs with less than 20 read counts across all samples, we used quantification of total bacterial load in the IgA and IgM sorted fractions to calculate the absolute abundance of a total of 1652 coated and 1766 strictly non-coated bacteria were identified across the sorted samples. For analysis of the quantitative differential abundance of the sorted fractions, we included ASVs based on a prevalence and abundance filtering stating that; if an ASV is present in at least five of the IgA or IgM sorted samples or in more than eight samples in the two study groups combined it is included or if the abundance is above 10^{-3} %.

Shotgun-based metagenomic sequencing and species identification

Metagenomic sequencing and species identification was done as described in Moll et al.[4]. Briefly, fecal bacterial DNA was extracted using the MetaHIT protocol as previously described[5]. Sequencing was performed using the BGISEQ-500 platform as described[6]. The integrated gene catalogue (IGC)[7] was clustered into 1507 metagenomic species containing at least 325 genes using the method described in Nielsen et al.[8] based on the gene abundance profiles across 2307 samples[7,9–12]. The metagenomic species were taxonomically annotated by summarizing gene annotations across all genes in a species. The genes were annotated by blasting the nucleotide sequences against RefSeq (downloaded on 1 October 2018) saving a maximum of 50 hits per gene with >= 45% sequence identity and >= 50% coverage. Species annotations from nucleotide BLAST were supplemented with annotations from protein BLAST (eukaryotic species only) and GTDBTk[13].

Single-nucleus sequencing analysis of intestinal plasma cells

Single-nucleus sequencing (snRNA-seq) data was acquired from a publicly available resource[14]. snRNA-seq data from 9 individuals was processed as described in the original paper[15]. In short, 9000 nuclei per sample was targeted for sequencing using Chromium Next GEM Single Cell 3' Reagent Kits v3.1 (10x Genomics). Raw snRNA-seq data processing was performed using the Cell Ranger Pipeline, https://support.10xgenomics.com/single-cell-gene-expression/software/pipelines/latest/what-is-cell-ranger) followed by quality control, dimensionality reduction and clustering using the Seurat's standard pipeline[16] and batch-corrected using RunHarmony[17]. Plasma cells were identified by Hickey et al. using the IGLL5 and AMPD1 marker genes. Plasma cells not expressing any of the IgG heavy chain genes was clustered as IgA+ and IgM+ plasma cells, while the remaining was clustered as IgG+ plasma cells. Average normalized expression was used for plotting Ig-expression levels at each intestinal segment.

References for Supplementary Materials and Methods

1. Callahan, B. J. et al. DADA2: High-resolution sample inference from Illumina amplicon data. *Nat Methods* **13**, 581-583 (2016). DOI: 10.1038/nmeth.3869
2. Quast, C. et al. The SILVA ribosomal RNA gene database project: improved data processing and web-based tools. *Nucleic Acids Res* **41**, D590-6 (2013). DOI: 10.1093/nar/gks1219
3. Bokulich, N. A. et al. Optimizing taxonomic classification of marker-gene amplicon sequences with QIIME 2's q2-feature-classifier plugin. *Microbiome* **6**, 90 (2018). DOI: 10.1186/s40168-018-0470-z
4. Moll, J. M. et al. Gut Microbiota Perturbation in IgA Deficiency Is Influenced by IgA-Autoantibody Status. *Gastroenterology* **160**, 2423-2434.e5 (2021). DOI: 10.1053/j.gastro.2021.02.053
5. Qin, J. et al. A metagenome-wide association study of gut microbiota in type 2 diabetes. *Nature* **490**, 55-60 (2012). DOI: 10.1038/nature11450

6. Fang, C. et al. Assessment of the cPAS-based BGISEQ-500 platform for metagenomic sequencing. *Gigascience* **7**, 1-8 (2018). DOI: 10.1093/gigascience/gix133

7. Li, J. et al. An integrated catalog of reference genes in the human gut microbiome. *Nat Biotechnol* **32**, 834-841 (2014). DOI: 10.1038/nbt.2942

8. Nielsen, H. B. et al. Identification and assembly of genomes and genetic elements in complex metagenomic samples without using reference genomes. *Nat Biotechnol* **32**, 822-828 (2014). DOI: 10.1038/nbt.2939

9. Karlsson, F. H. et al. Gut metagenome in European women with normal, impaired and diabetic glucose control. *Nature* **498**, 99-103 (2013). DOI: 10.1038/nature12198

10. Bäckhed, F. et al. Dynamics and Stabilization of the Human Gut Microbiome during the First Year of Life. *Cell Host Microbe* **17**, 690-703 (2015). DOI: 10.1016/j.chom.2015.04.004

11. Nakatsu, G. et al. Gut mucosal microbiome across stages of colorectal carcinogenesis. *Nat Commun* **6**, 8727 (2015). DOI: 10.1038/ncomms9727

12. Yu, J. et al. Metagenomic analysis of faecal microbiome as a tool towards targeted non-invasive biomarkers for colorectal cancer. *Gut* **66**, 70-78 (2017). DOI: 10.1136/gutjnl-2015-309800

13. Parks, D. H. et al. A standardized bacterial taxonomy based on genome phylogeny substantially revises the tree of life. *Nat Biotechnol* **36**, 996-1004 (2018). DOI: 10.1038/nbt.4229

14. Hickey, J. et al. High Resolution Single Cell Maps Reveals Distinct Cell Organization and Function Across Different Regions of the Human Intestine [Internet]. *HuBMAP Consortium* (2022). DOI: 10.35079/HBM692.JRZB.356

15. Hickey, J. W. et al. Organization of the human intestine at single-cell resolution. *Nature* **619**, 572-584 (2023). DOI: 10.1038/s41586-023-05915-x

16. Stuart, T. et al. Comprehensive Integration of Single-Cell Data. *Cell* **177**, 1888-1902.e21 (2019). DOI: 10.1016/j.cell.2019.05.031

17. Korsunsky, I. et al. Fast, sensitive and accurate integration of single-cell data with Harmony. *Nat Methods* **16**, 1289-1296 (2019). DOI: 10.1038/s41592-019-0619-0
