# Supplementary material for: IgG and IgM cooperate in coating of intestinal bacteria in IgA deficiency
Source: Nat Commun. 2023 Dec 8;14:8124. doi: 10.1038/s41467-023-44007-2 (PMC10709418; doi:10.1038/s41467-023-44007-2)
Supplement: Supplementary file 3 — Description of Additional Supplementary Files [file 41467_2023_44007_MOESM3_ESM.pdf]

Description of Additional Supplementary Files

File Name: Supplementary Data 1
Description: Mean count of ASVs found in control samples.

File Name: Supplementary Data 2
Description: Bacterial abundances of sorted bacteria coated with IgA or IgM in household-matched IgA+ or IgA- subjects.

File Name: Supplementary Data 3
Description: Bacterial abundances of non-coated sorted bacteria in household-matched IgA+ or IgA- subjects.
