# Supplementary material for: IgG and IgM cooperate in coating of intestinal bacteria in IgA deficiency
Source: Nat Commun. 2023 Dec 8;14:8124. doi: 10.1038/s41467-023-44007-2 (PMC10709418; doi:10.1038/s41467-023-44007-2)
Supplement: Supplementary file 7 — Reporting Summary [file 41467_2023_44007_MOESM7_ESM.pdf]

nature portfolio

Corresponding author(s): Brix, S
Last updated by author(s): Nov 6, 2023

Reporting Summary

Nature Portfolio wishes to improve the reproducibility of the work that we publish. This form provides structure for consistency and transparency in reporting. For further information on Nature Portfolio policies, see our Editorial Policies and the Editorial Policy Checklist.

Statistics

For all statistical analyses, confirm that the following items are present in the figure legend, table legend, main text, or Methods section.

n/a	Confirmed	
☐	☒	The exact sample size (*n*) for each experimental group/condition, given as a discrete number and unit of measurement
☐	☒	A statement on whether measurements were taken from distinct samples or whether the same sample was measured repeatedly
☐	☒	The statistical test(s) used AND whether they are one- or two-sided<br>*Only common tests should be described solely by name; describe more complex techniques in the Methods section.*
☐	☒	A description of all covariates tested
☐	☒	A description of any assumptions or corrections, such as tests of normality and adjustment for multiple comparisons
☐	☒	A full description of the statistical parameters including central tendency (e.g. means) or other basic estimates (e.g. regression coefficient) AND variation (e.g. standard deviation) or associated estimates of uncertainty (e.g. confidence intervals)
☐	☒	For null hypothesis testing, the test statistic (e.g. *F*, *t*, *r*) with confidence intervals, effect sizes, degrees of freedom and *P* value noted<br>*Give P values as exact values whenever suitable.*
☒	☐	For Bayesian analysis, information on the choice of priors and Markov chain Monte Carlo settings
☒	☐	For hierarchical and complex designs, identification of the appropriate level for tests and full reporting of outcomes
☒	☐	Estimates of effect sizes (e.g. Cohen's *d*, Pearson's *r*), indicating how they were calculated

Our web collection on statistics for biologists contains articles on many of the points above.

Software and code

Policy information about availability of computer code

Data collection	BD FACSDiva Software v. 6.1.3 was used to collect the flow cytometry data.
Data analysis	R v3.5.1 was used for statistical analyses and data visualization.<br>BBTools v38.37 was used to remove sequencing adaptors.<br>MAFFT v. 7.4.53 (standard settings) was used for alignments.<br>FastTree v. 2.1.10 was used for generation of phylogenetic trees<br>DADA2 v. 1.10 was used to denoise and analyze sequencing reads.<br>GhostKOALA v. 2.1 (https://www.kegg.jp/ghostkoala) was used for identification of KEGG orthology groups.<br>FlowJo v. 10.5.0 From Tree Star was used for analysis of flow cytometry data.

For manuscripts utilizing custom algorithms or software that are central to the research but not yet described in published literature, software must be made available to editors and reviewers. We strongly encourage code deposition in a community repository (e.g. GitHub). See the Nature Portfolio guidelines for submitting code & software for further information.

Data

Policy information about availability of data

All manuscripts must include a data availability statement. This statement should provide the following information, where applicable:

- Accession codes, unique identifiers, or web links for publicly available datasets
- A description of any restrictions on data availability
- For clinical datasets or third party data, please ensure that the statement adheres to our policy

Source data are provided with this paper. The raw nucleotide datasets have been deposited ingenerated during the current study are available in the European Nucleotide Archive repository under accession number: PRJEB55323 [https://www.ebi.ac.uk/ena/browser/view/PRJEB55323] or National Center for Biotechnology Information Sequence Read Archive (SRA) under accession number: PRJNA633381 [https://www.ncbi.nlm.nih.gov/bioproject/PRJNA633381]. Sequence data used to identify mucus-embedded bacteria are deposited in the European Nucleotide Archive under accession number: PRJEB28097 [https://www.ebi.ac.uk/ena/browser/view/PRJEB28097]. The fastq-files from the FACS-sorted samples are available upon request. Assignment of taxonomy was done using the SILVA database v. 132 [https://doi.org/10.5281/zenodo.1172783]. Other non-GDPR restricted data generated during and/or analyzed during the current study are available from the corresponding author on reasonable request. Individual-level personally identifiable data from the individuals participating in the cohort cannot be made freely available, to protect the privacy of the participants, in accordance with the Danish Data Protection Act and European Regulation 2016/679 of the European Parliament and of the Council (GDPR) that prohibit distribution even in pseudo-anonymized form. However, research collaborations are welcome, and data can be made available under a joint research collaboration by contacting corresponding author Susanne Brix (sbrix@dtu.dk).

Research involving human participants, their data, or biological material

Policy information about studies with human participants or human data. See also policy information about sex, gender (identity/presentation), and sexual orientation and race, ethnicity and racism.

Reporting on sex and gender	Since the study is designed in a way where each IgA deficient subject is matched with a household member for better adjustment for factors like place of living, diet and other environmental variables, each IgA deficient subject will most often be matched in a way where subjects with a female sex will be matched with subjects with a male sex. The distribution is shown below under population characteristics.
Reporting on race, ethnicity, or other socially relevant groupings	Race, ethnicity or other socially groupings was not reported as part of the presented study.
Population characteristics	Sex:<br>IgA deficient: 20 female, 11 male<br>IgA sufficient household members: 12 female, 18 male, 1 unknown<br><br>Age (Mean±SD):<br>IgA deficient: 57.5±14.7<br>IgA sufficient household members: 57.4±15.9
Recruitment	Samples were collected at the Department of Clinical Immunology at the Karolinska University Hospital, Huddinge, Sweden. Individuals with IgA deficiency (serum IgA of <0.07 g/L) living in the Stockholm area were invited to take part in the current study. Subjects with IgAD (no antibiotics for >60 days) and their partners (sharing households and an omnivorous diet with no yogurt products >5 days before sampling), with no reported or ongoing gastrointestinal problems (inflammatory bowel disease, celiac disease, or lactose intolerance) were included in the study after informed consent, and fecal and blood samples were collected.
Ethics oversight	The study was approved by regional ethical committee, Stockholm, Sweden (No: 2016/2502-31/2). Samples for comparison to single-IgG2 coating prevalence in Crohn's disease patients were derived from the IBD South Limburg (IBDSL) cohort, which is a population-based inception cohort from the South Limburg area of the Netherlands, collected as described (PMID: 26045509), approved by the local Medical Ethics Committee, and registered in http://www.clinicaltrials.gov (NCT02130349).

Note that full information on the approval of the study protocol must also be provided in the manuscript.

Field-specific reporting

Please select the one below that is the best fit for your research. If you are not sure, read the appropriate sections before making your selection.

☒ Life sciences ☐ Behavioural & social sciences ☐ Ecological, evolutionary & environmental sciences

For a reference copy of the document with all sections, see nature.com/documents/nr-reporting-summary-flat.pdf

Life sciences study design

All studies must disclose on these points even when the disclosure is negative.

Sample size	No sample size calculations were performed as part of the study. The number of individuals enrolled in the study was based on the availability

Sample size	of paired IgA deficient/IgA sufficient household member samples from the cohort.
Data exclusions	No data were excluded.
Replication	We included controls to compensate for any potential day to day variation, including blank DNA extraction and PCR controls, and an internal control sample. Reproducibility of the Ig coating pattern was successfully replicated in all attempts assessed by the use of the internal control included each analysis day (n = 16).
Randomization	IgA deficient individuals and corresponding household member samples were handled together to maintain the paired nature of the setup. Each pair of samples were randomized throughout the study i.e. during flow cytometry analysis, DNA extraction, library preparation etc.
Blinding	The paired setup prevented the investigators from being fully blinded, but otherwise all analyses were conducted in a blinded manner.

Reporting for specific materials, systems and methods

We require information from authors about some types of materials, experimental systems and methods used in many studies. Here, indicate whether each material, system or method listed is relevant to your study. If you are not sure if a list item applies to your research, read the appropriate section before selecting a response.

Materials & experimental systems

n/a	Involved in the study	
☐	☒	Antibodies
☒	☐	Eukaryotic cell lines
☒	☐	Palaeontology and archaeology
☒	☐	Animals and other organisms
☒	☐	Clinical data
☒	☐	Dual use research of concern
☒	☐	Plants

Methods

n/a	Involved in the study	
☒	☐	ChIP-seq
☐	☒	Flow cytometry
☒	☐	MRI-based neuroimaging

Antibodies

Antibodies used	Flow cytometry:<br>Anti-Human IgA-PE, Provider: Miltenyi Biotec Cat no: 130-093-128 clone: IS11-8E10<br>Anti-Human IgM-APC/Cy7, Provider: Biolegend Cat no: 314520 clone: MHM-88<br>Anti-Human IgG-PE/Cy7, Provider: Biolegend Cat no: 409316, clone: HP6017<br>Anti-Human IgG1-Biotin + Streptavidin-APC-Cy7, Provider: SouthernBiotech Cat no: 9054-28, clone: HP6001,<br>Anti-Human IgG2-AF647, Provider: SouthernBiotech Cat no: 9070-31, clone: HP6002<br>Anti-Human IgG3-AF488, Provider: SouthernBiotech Cat no: 9210-30, clone: HP6050<br>Anti-Human IgG4-PE, Provider: SouthernBiotech Cat no: 9200-09, clone: HP6025<br>ELISA:<br>Anti-Human IgG-HRP, Provider: SouthernBiotech Cat no: 0150-05, clone: polyclonal<br>Anti-Human IgG1 Fc-UNLB, Provider: SouthernBiotech Cat no: 9054-01, clone: HP6001<br>Anti-Human IgG2 Fc-UNLB, Provider: SouthernBiotech Cat no: 9070-01, clone: HP6002<br>Anti-Human IgG3 Fc-UNLB, Provider: SouthernBiotech Cat no: 9210-01, clone: HP6050<br>Anti-Human IgG4 Fc-UNLB, Provider: SouthernBiotech Cat no: 9200-01, clone: HP6025
Validation	Each antibody used for flow cytometry was tested and validated using FMO, as shown in Supplementary figure S1 and S2.<br>All antibodies were from the commercial sources listed above. The validation statement for flow cytometry or ELISA are available on the manufacturer's website.

Flow Cytometry

Plots

Confirm that:

☒ The axis labels state the marker and fluorochrome used (e.g. CD4-FITC).

☒ The axis scales are clearly visible. Include numbers along axes only for bottom left plot of group (a 'group' is an analysis of identical markers).

☒ All plots are contour plots with outliers or pseudocolor plots.

☒ A numerical value for number of cells or percentage (with statistics) is provided.

Methodology

Sample preparation	Fecal samples were immediately frozen, transferred to the laboratory on dry ice, and kept frozen until processed as described in the method section of the manuscript.

Instrument	Ig-coating frequencies and bacterial counting was performed on a FACSCanto II flow cytometer (BD Biosciences)<br>Bacterial sorting was performed on a MoFlo XDP cell sorter (Beckman Coulter).
Software	The flow cytometry data were collected using the BD FACSDiva Software v. 6.1.3.<br>Flow cytometry data were analyzed using FlowJo (Version 10.5.0, Tree Star, Inc., Ashland, OR).
Cell population abundance	For each of the sorted populations of either total IgA-coated, total IgM-coated bacteria or uncoated bacteria, we sorted out an average of 2x10^6 bacteria that were further processed for 16S rRNA gene amplicon sequencing.
Gating strategy	The samples were gated on the DAPI+ population used to separate bacteria from debris and next gated on each flourochrome as shown in Figure 1a & Supplementary figure 1 and 2.

☒ Tick this box to confirm that a figure exemplifying the gating strategy is provided in the Supplementary Information.
